# Total aortic arch debranching with antegrade Thoracic Endovascular Aortic Repair (TEVAR) in acute non-A non-B aortic dissection

**DOI:** 10.1186/s13019-024-02917-2

**Published:** 2024-06-28

**Authors:** Aaron Gilani, Benjamin Schachner, Elizabeth Wood, Zohaib Khawaja, Bartlomiej Imielski

**Affiliations:** Department of Cardiothoracic Surgery, Medical Center Boulevard, Atrium Health Wake Forest Baptist, Winston-Salem, NC 27157 USA

**Keywords:** TEVAR, High-risk surgical repair, Debranching, Aortic dissection, Hybrid therapy

## Abstract

**Background:**

The surgical evaluation and management of non-A non-B aortic dissections, in the absence of ascending aortic involvement, remains a grey area. It is in these scenarios when thorough evaluation of patient/family history, clinical presentation, but also overall lifestyle, is of immense importance when determining an optimal intervention.

**Case presentation:**

We present a 38-year-old patient with a physically demanding lifestyle as a professional wrestler, uncontrolled hypertension due to history of medical non-adherence, and family history of aortic dissection who presented with acute non-A non-B aortic dissection. He was spared a total arch replacement by undergoing a hybrid approach of complete aortic debranching with antegrade Thoracic Endovascular Aortic Repair (TEVAR). The patient was able to benefit from reduced cardiopulmonary bypass (CPB) time, avoidance of aortic cross clamp, circulatory arrest, and hypothermic circulation.

**Conclusions:**

This patient’s unique composition of a physically demanding lifestyle, personal history of medical non-adherence, family history of aortic dissection, and clinical presentation required a holistic approach to understanding an ideal intervention that would be best suited long-term. Due to this contextualization, the patient was able to be spared a total arch replacement, or suboptimal medical management, by instead undergoing a hybrid-approach with total aortic arch debranching with antegrade TEVAR.

## Background

While aortic dissections are largely divided into Types A and B based on the Stanford Classification, non-A non-B aortic dissections with intimal tears extending between the innominate artery and left subclavian artery are a more controversial area regarding their classification and surgical management [1]. It is in these scenarios that synthesis and contextualization of all available clinical information is of paramount importance. It is in these patients that a hybrid approach using both open surgical aortic debranching and Thoracic Endovascular Aortic Repair (TEVAR) can be of additive value while decreasing exposure to risks associated with longer CPB times, total aortic cross-clamping, circulatory arrest, and hypothermic circulation.

In this report, we document the management of a young patient with non-A non-B aortic dissection by utilizing a hybrid approach of aortic arch debranching and TEVAR given his physically-demanding lifestyle as a professional wrestler, failed medical therapy for hypertension, family history of early-age aortic dissection, as well as consideration of his clinical anatomy and pathophysiology. Informed consent was obtained from the patient regarding publishing of case details and images.

### Case presentation

**Fig. 1:**
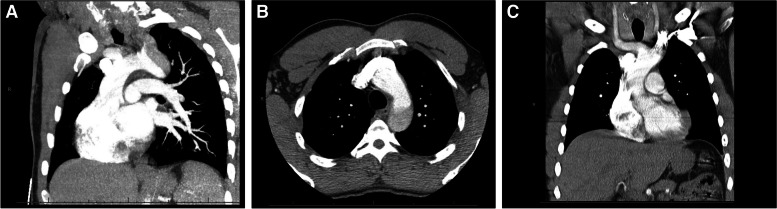
**A-C** Initial presentation CT angiogram with Zone 3 aortic dissection with retrograde extension into left subclavian artery (1A: Sagittal cross-section; 1B: Axial cross-section; 1C: Coronal cross-section)

A 38-year-old high-performance athlete (height: 1.78 m; weight: 76 kg) with a past medical history of uncontrolled hypertension, no prior anticoagulation or antiplatelet therapy, and family history of first-degree relative with aortic dissection at similar young age presented to outside hospital with four days of chest pain. Patient underwent subsequent CT chest which revealed intimal tear in Zone 3 of the aorta with dissection into the left subclavian artery, with a distance of 3.5 mm from the primary entry tear to the left subclavian artery (Fig. 1A-C: Initial presentation CT angiogram with Zone 3 aortic dissection with retrograde extension into left subclavian artery; 1A: Sagittal cross-section; 1B: Axial cross-section; 1C: Coronal cross-section). Following this finding, the patient was emergently transferred to a nearby hospital for operative assessment and intervention.

**Fig. 2:**
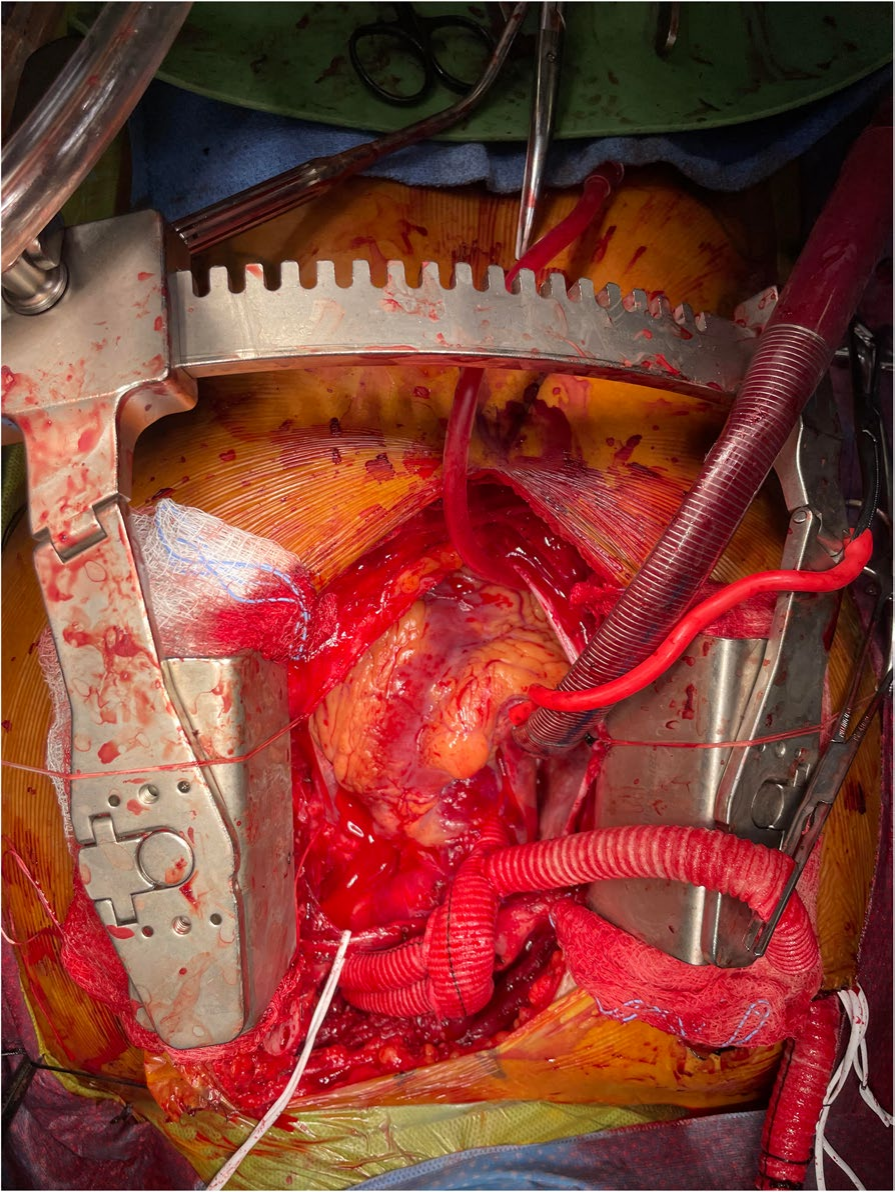
Intraoperative photograph following placement of grafts

Upon arrival and evaluation of patient, he was taken to operating room with plan for total aortic arch debranching with antegrade TEVAR. Intra-operative transesophageal echocardiogram (TEE) revealed preserved left-ventricular ejection fraction of 55% with insignificant valvular dysfunction. The patient underwent full conventional sternotomy with 14 mm Hemashield Gold trifurcated graft to establish flow to innominate, left subclavian, and left common carotid arteries, as well as antegrade TEVAR placement, via a fourth sewn on 10 mm graft, using a 34 mm x 15 mm Gore thoracic aortic endograft (Fig. 2: Intraoperative photograph of graft placement). Only partial aortic cross-clamp was used of the non-dissected ascending aorta, the heart was not arrested, and total CPB time was 187 min. Post-operative TEE revealed LVEF of 55% with borderline normal right ventricular function.

**Fig. 3:**
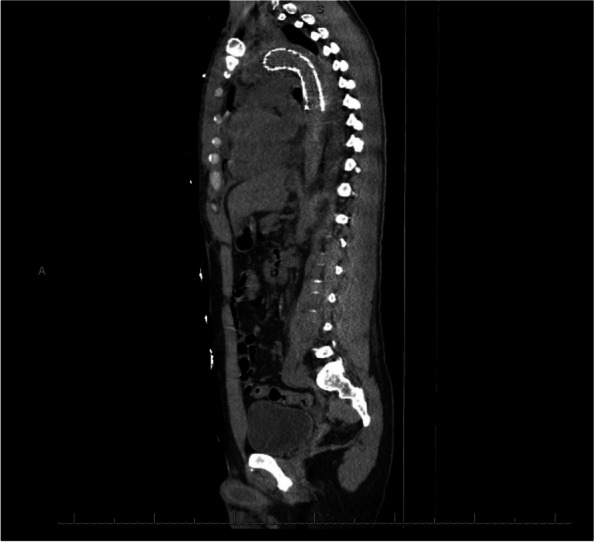
Post-operative day 4 CT angiogram showing positioning of 34 mm x 15 mm Gore thoracic endograft (sagittal cross-section)

After the operation, the patient recovered in the Cardiovascular Intensive Care Unit (CVICU) where he was weaned off vasopressor support, extubated on post-operative day one without complication, and transferred to the floor on post-operative day two. On post-operative day four, the patient underwent CT angiogram of the chest, abdomen, and pelvis to evaluate the graft which revealed improving false lumen opacification at the grafted aorta (Fig. 3: Post-operative day 4 CT angiogram showing positioning of 34 mm x 15 mm Gore thoracic endograft; sagittal cross-section). The remainder of his post-operative course was unremarkable, and the patient was discharged home on post-operative day six with oral antihypertensive regimen, Aspirin 81 mg daily as antiplatelet therapy, and extensive counseling.

## Discussion/Conclusions

In patients with physically demanding lifestyle, history of failed medical therapy, and/or family history of early onset aortic dissection, a holistic approach to pre-operative planning is essential, whilst maintaining the urgency associated with the pathophysiology of an acute non-A non-B aortic dissection. In this patient, we were able to identify three key inflection points involving our decision-making process: validity of conservative management, appropriateness of TEVAR-only intervention, feasibility of total arch replacement.

Hypertension is considered a key risk factor for acute aortic dissection. Hypertensive patients with acute aortic dissections have had a longer history, higher stage, worse medication compliance, and poor control of hypertension [2]. Additionally, poor compliance was associated with increased healthcare costs to the patient [3]. Unfortunately, this patient’s history of poor medical compliance, and subsequently uncontrolled hypertension, made him less-than-suitable for medical therapy alone. Additionally, performance athletes engaged in rigorous weight-training can have acute rises in systolic blood pressure to 300 mmHg, further increasing risk of development of acute aortic dissection [4]. As such, this patient’s baseline physically demanding lifestyle as a performance athlete leaves him vulnerable to wide variations in his blood pressure in the absence of optimal control.

The patient’s family history was also notable for first-degree relative with a Stanford Type A aortic dissection at a young age. Elucidating a thorough history in patients presenting with acute aortic dissection, even in the absence of a known genetic mutation, is of a great importance given that non-syndromic familial thoracic aortic dissections are inherited in an autosomal dominant pattern with variable age of disease onset [5].

This patient also posed a variety of anatomic and physiologic considerations. Given insufficient proximal landing zone for a TEVAR-only approach, and lack of need for conventional total arch replacement given absence of ascending aortic dissection or intramural hematoma, the hybrid utilization of aortic debranching and TEVAR was advantageous in multiple areas. The hybrid approach allowed for shorter CPB bypass time (187 min) versus average CPB for total aortic arch replacement of 241 min [6]. A shorter CPB is associated with longer duration of ventilation, longer CVICU stay, and longer overall hospital stay [7]. Additionally, given this hybrid approach, the patient did not require total aortic cross clamp or circulatory arrest. Prolonged aortic cross clamp is associated with low cardiac output, prolonged ventilation, renal complication, blood transfusion, mortality and prolonged hospital stay [8]. Patients undergoing total arch replacement also require utilization of hypothermic circulation for prevention of organ ischemia, of which approximately 15% suffer the sequela of post-operative hypothermia [9]. We were able to avoid the need for hypothermic circulation given the utilization of the hybrid debranching and TEVAR technique. Additionally, this method utilized minimal anastomoses, therefore leaving the patient less susceptible to bleeding from anastomotic sites and a potentially decreased need for transfusion and its subsequently associated complications.

Management of acute non-A non-B aortic dissections remains infrequently documented, as well as a fluid area of management which requires thorough, yet expeditious, evaluation of a patient’s clinical picture [10]. Particularly, our patient case provided a number of considerations including elevated baseline physiological demand as a performance athlete, history of medical therapy non-adherence, and presence of family history of aortic dissection, which made our patient a suitable candidate for hybrid technique utilizing total aortic debranching and antegrade TEVAR.

## Data Availability

No datasets were generated or analysed during the current study.
